# Exploring Community-Dwelling Older Adults’ Considerations About Values and Preferences for Future End-of-Life Care: A Study from Sweden

**DOI:** 10.1093/geront/gnaa012

**Published:** 2020-03-21

**Authors:** Malin Eneslätt, Gert Helgesson, Carol Tishelman

**Affiliations:** 1 Department of Learning, Informatics, Management and Ethics, Karolinska Institutet, Stockholm, Sweden; 2 Department of Health Sciences, Luleå University of Technology, Sweden; 3 Center for Rural Medicine (GMC), Storuman, Sweden; 4 Stockholm Health Care Services (SLSO), Region Stockholm, Sweden; 5 School of Health Sciences, University of Southampton, UK

**Keywords:** Advance care planning, Go Wish, Qualitative research methods, End-of-life care, DöBra

## Abstract

**Background and Objectives:**

There is a substantial body of research on advance care planning (ACP), often originating from English-speaking countries and focused on health care settings. However, studies of content of ACP conversations in community settings remain scarce. We therefore explore community-dwelling, older adults’ reasoning about end-of-life (EoL) values and preferences in ACP conversations.

**Research Design and Methods:**

In this participatory action research project, planned and conducted in collaboration with national community-based organizations, we interviewed 65 older adults without known EoL care needs, about their values and preferences for future EoL care. Conversations were stimulated by sorting and ranking statements in a Swedish version of GoWish cards, called the DöBra cards, and verbatim transcripts were analyzed inductively.

**Results:**

While participants shared some common preferences about EoL care, there was great variation among individuals in how they reasoned. Although EoL preferences and prioritizations could be identical, different individuals explained these choices very differently. We exemplify this variation using data from four participants who discussed their respective EoL preferences by focusing on either physical, social, existential, or practical implications.

**Discussion and Implications:**

A previously undocumented benefit of the GoWish/DöBra cards is how the flexibility of the card statements support substantial discussion of an individual’s EoL preferences and underlying values. Such in-depth descriptions of participants’ reasoning and considerations are important for understanding the very individual nature of prioritizing EoL preferences. We suggest future users of the DöBra/GoWish cards consider the underlying reasoning of individuals’ prioritizations to strengthen person-centeredness in EoL conversations and care provision.

Advance care planning (ACP) is an umbrella term for a process of reflection and planning for future end-of-life (EoL) care that may include discussions with others and written documentation of preferences in an advance directive (Rietjens et al., 2017; The International Society of Advance Care Planning, 2018). ACP has been associated with increased documentation of EoL care preferences, improved EoL communication, and increased proportion of deaths occurring in a preferred place (Jimenez et al., 2018). Definitions of ACP limited to the completion of advance directives have been criticized with arguments that complex conversation-based ACP interventions are more effective than written documentation alone (Brinkman-Stoppelenburg, Rietjens, & van der Heide, 2014). Jimenez and colleagues (2018) suggest that effective ACP programs should include repeated and interactive discussion sessions, decision aids, and interventions targeting multiple stakeholders at individual, organizational, and policy levels.

Recent reviews of older peoples’ and seriously ill individuals’ experiences of engaging in ACP found varied and often ambivalent feelings towards participation (Ke, Huang, Hu, O’Connor, & Lee, 2017; Zwakman et al., 2018). ACP was generally said to be experienced as informative, helpful for planning future care, and reducing risk of future conflicts in families. It could, however, also be seen as distressing, as not reality-based because there is no guarantee that preferences will be fulfilled, and as potentially confrontational in having to face your own death. Zwakman and colleagues (2018) found that participants with life-limiting illnesses suggested that ACP should take place sooner rather than later, to be experienced as less confrontational and therefore easier to accept. Recent studies have pointed to benefits in designing early, community-based interventions targeting people prior to diagnosis with life-limiting illnesses (Abba, Lloyd-Williams, & Horton, 2019; Banner et al., 2019; Costello, Hinderer, Lee, & Chike-Udegha, 2015).

Although advance directives are briefly mentioned in the Swedish National Guidelines for Palliative Care (Regionalt cancercentrum Stockholm Gotland, 2016), ACP is not systematically practiced in Sweden today, and there are no legal grounds for formulating binding advance directives in the Swedish system. While individual consideration of future EoL issues is also likely to take place in Sweden, the lack of systematic practice motivates consideration of the Swedish context itself as ACP-naive on a collective level. Based on previous international research and adapted to the Swedish context, the approach to ACP in this research project: “Advance care planning in Sweden” (SweACP), is based on three cornerstones as it is (a) conversation-based (Sudore & Fried, 2010; Vearrier, 2016), (b) initiated early (Howard et al., 2015; Zwakman et al., 2018), and (c) community-based, that is, taking place outside the health and social care systems (Litzelman et al., 2017; Somes et al., 2018).

There is an extensive body of research on ACP, particularly from English-speaking countries, for example, on ACP interventions and programs (Carter et al., 2018; Sudore, Schillinger, et al., 2018), facilitators’ and target persons’ experiences of engaging in ACP (Howard et al., 2018; Somes et al., 2018), and ACP in different minority populations (Laury, Mackenzie-Greenle, & Meghani, 2019; Sudore, Cuervo, et al., 2018). However, surprisingly little has been published about the actual content of ACP conversations and how values and preferences are discussed by individuals (Hirakawa, Chiang, Hilawe, & Aoyama, 2017; Robinson, Hart, & Sanders, 2019). Such knowledge can be valuable in supporting a reflective ACP process which can further person-centered care. We aim to address this knowledge gap by exploring how community-dwelling, older adults reason about their values and preferences for future EoL care in ACP conversations.

## Design and Methods

### Study Context

In Sweden, specialized palliative care reaches between 3% and 22% of those dying depending on diagnosis, region, and place of care (The Swedish Register of Palliative Care, 2017). Most deaths occur in institutional settings, with about 40% each in acute care hospitals and in residential care homes (Håkanson, Öhlén, Morin, & Cohen, 2015). A survey of the general public (Westerlund et al., 2018) found limited awareness of issues related to EoL, which impinges on people’s ability to engage in ACP and advocate for quality EoL care. The survey showed numerous descriptions of hinders along with an expressed need for competency building and collaboration across individuals, groups, and communities to support engagement in EoL issues (Westerlund et al., 2018). This inspired development of the Swedish *DöBra*^1^ research program, which uses new public health and participatory action research approaches with the long-term goal of promoting constructive change and contributing to diminishing avoidable suffering related to dying, death, and bereavement.

Data presented here derive from one component of this comprehensive research program, called SweACP. SweACP is a nation-wide research project, planned and conducted in active collaboration among researchers with a variety of disciplinary and professional backgrounds (ethics, ethnology, gerontology, nursing, palliative care, philosophy, social psychology) and a range of national, community-based interest and patient organizations: the Association of Relatives to Cancer Patients, the Dementia Association, the Lung Cancer Interest Organization, Network against Cancer, the Swedish Association for Senior Citizens, and the Swedish National Pensioners’ Organization (PRO).

### Recruitment

We were interested in recruiting people from the community without imminent EoL care needs. As ACP is not practiced in Sweden, and there is limited public discussion of EoL issues, we were reluctant to predetermine a sample and risk unexpectedly confronting potential participants with issues related to death and dying to which they would have to respond. After obtaining ethical approval (Stockholm #2015/106–31/5), we therefore used purposeful sampling and a recruitment process based on active volunteering, in collaboration with the above-named community organizations (Tishelman, Eneslätt, Menkin, & Lindqvist, 2019). Information about the project was presented in several organizations’ membership newsletters and at membership meetings of local chapters of PRO, with instructions to contact the researchers for more information if interested in participating. From May 2015 to December 2016, 77 people took an active stance in volunteering through contacting the researchers. Eleven potential participants were not interviewed, due to logistical problems in finding a suitable date or place to meet (*n* = 4); declining after learning more about the study (*n* = 4); unreachable by phone/e-mail after initial contact (*n* = 2); or hospitalization (*n* = 1). Most participants were recruited nationwide through PRO (*n* = 25) and the Dementia Association (*n* = 23), and a few through other collaborating community organizations (*n* = 5). Others were enrolled through spontaneous snowball recruitment as individuals who had heard about the study from prior participants also contacted us asking to participate (*n* = 13).

### Data Collection

Sixty-one interviews were conducted with 66 people (five interviews with couples) from May 2015 to January 2017. The data from one interview were later excluded as we noted during the interview that the severity of the participant’s cognitive impairment might hinder appropriate understanding of informed consent, resulting in a database of 60 interviews with 65 people.

After receiving informed consent, all interviews were conducted by one of the two female research assistants (M.E. and T.J.) previously unknown to participants. Both had prior experience of conducting research interviews but had different disciplinary backgrounds (MSc in nursing [M.E.] and applied forensic psychology [T.J.]). Two interviews were conducted by both interviewers together to enhance consistency. Interviews were held at a place of each participant’s choice, typically in their homes but also at various more public venues, and ranged from 30 to 133 minutes, generally lasting about an hour. The audio-recorded interviews were held in conversational form and were supported by an interview guide with topics to cover and probing questions. The protocol had been previously tested in group discussions with volunteering representatives of the collaborating organizations (Tishelman et al., 2019).

The first section of the interview consisted of an open conversation, stimulated by questions on *who* and *what* would be important to participants in their future EoL, with interviewers probing for detailed descriptions. The term EoL was not predefined by researchers, but participants discussed it as the last months, weeks, or days of life, depending on disease trajectory. The second interview section was semistructured, beginning with a graphic depiction of *who* would be important at the EoL (i.e., participants’ social networks; beyond the scope of this article and reported on elsewhere (Tishelman et al., 2019)). Thereafter, the translated and adapted Swedish version of GoWish cards (Menkin, 2007), the DöBra cards, were used to reflect on and discuss *what* was important, in terms of values and preferences for future EoL care (see Tishelman et al., 2019 for description of development and initial testing of this protocol). A final summarizing section included reflections about participating in the ACP conversation. Participants were also asked for demographic information and responded to a global question about their self-rated health status with three alternatives; good, neither good nor bad, or bad. See Supplementary Material for full listing of the procedure.

The interviews with couples followed the same protocol as individual interviews, with the distinction that partners in the couples often complemented and contrasted each other’s answers when conversing and could ask further questions of one another. Each person in the couple sorted and ranked the DöBra cards individually, generally one person at a time in an order of their choice, with ongoing discussion.

The English-language GoWish cards were originally developed in the United States (Menkin, 2007), based on Steinhauser and colleagues’ research (2000) about EoL issues deemed most important by different stakeholder groups. The cards were, with granted permission from the originators (www.codaalliance.org), translated, and adapted to the Swedish context by the project group collaboratively in a series of meetings (Tishelman et al., 2019). The finalized Swedish DöBra cards consist of 37 preformulated items of potential importance at the EoL, for example, “*to be free of pain,”* “*not dying alone,”* “*to have my financial affairs in order*,” *and “to pray.”* In addition, there are three “wild cards” for matters of individual importance not covered by the preformulated items (see Supplementary Material for full listing of items).

Participants were asked to sort each DöBra card into one of three piles according to its priority—very important, somewhat important, and not important—and were encouraged to express their reflections and reasoning. The conversation continued with further focus on the card items deemed very important, and the 10 most important cards were ranked from 1 to 10, with the first most important. This is the procedure originally suggested for GoWish (Menkin, 2007) and used by other researchers (Lankarani-Fard et al., 2010; Litzelman et al., 2017). Participants were given the card deck to keep if they so desired after the interview, but no other reimbursement was offered. Brief field notes were taken by interviewers during interviews, and a longer reflective summary was formulated after each interview. Field notes documented features of interviews not captured by audio-recordings, for example, participant reactions, setting, etc. and were used to inform later analysis. The audio-recordings of the interviews were professionally transcribed verbatim, and transcripts were checked for errors by the responsible interviewer who also added comments from field notes.

### Data Analysis

The analysis presented here has been a process marked by repeated “false starts” as we initially strived to find common themes in data we later found to be characterized by great individual variation. On a conceptual level, analysis was inspired by Interpretive Description (Thorne, 2016) and performed as detailed below. The analysis was carried out primarily by the first author (M.E.), a doctoral student and RN, with input from the coauthors who both hold PhDs and have backgrounds in nursing (C.T.) and philosophy/ethics (G.H.). Analysis and manuscript drafts have also been discussed with T.J. and other research colleagues familiar with the DöBra cards but not involved in this project.

M.E., who had conducted 45 of the interviews, listened and reread the full database comprised of all 60 interviews with 65 participants in an effort to gain an initial understanding of the content of each and the data as a whole. Given the unusually large database for qualitative analysis, necessary for other aspects of the SweACP project (Tishelman et al., 2019), a process of stepwise purposive sampling (Flick, 2014; Forss, Tishelman, Widmark, & Sachs, 2004) was undertaken in which a subsample of interviews was selected for more in-depth analysis based on the following criteria. First, eight interviews deemed as deviant or outliers in relation to reasoning, experiences, or opinions were included. These were complemented with four additional interviews, chosen to achieve maximal variation based on demographic features. Given the lack of guidance in the extant literature, we chose features we considered potentially could affect the participants’ perspectives, that is, age, sex, place of residence (rural/urban), living situation, and self-reported health status. After initial analysis of these 12 interviews, we included six additional interviews in which experiences and opinions seemed to be in line with those in many other interviews, thus considered more “typical” for the full database. These were chosen to provide further in-depth knowledge in relation to the study aim (see also Malterud, Siersma, and Guassora [2016]). Repeated reading of these 18 interviews with 22 respondents (four interviews with couples) was followed by a process in which data were sorted in the NVivo software program by interview section, that is, whether they derived from the initial open conversation or the later semistructured component using the DöBra cards to allow understanding of the context. An initial iterative process involving all authors included inductively sorting data into patterns (Thorne, 2016) to derive preliminary common themes, including “Anticipating loss of independence” and “Wishing to control the time-point of death.” However, we could not see that this form of thematization contributed new knowledge to the field and, more importantly, did not adequately represent the content of the data set.

We became aware that there were individual patterns of reasoning which we failed to adequately understand in this manner, but which became the focus of continued analysis and the presentation of results below. Instead of continuing to organize data by themes based on similarities, we began to explore patterns in rankings of prioritized DöBra cards by individual participants. This step was inspired by the categorization of the GoWish card statements into physical, practical, existential, and social dimensions documented by Osman, El Jurdi, Sabra, and Arawi (2018). This contributed to a new perspective in data analysis, as the great individual variation in rankings was striking and we therefore continued to explore differences among participants in how they reasoned.

In a next step, we chose a new subsample for analysis, for two reasons. We wanted to revisit our analysis to ensure it was robust and sought a clear way of presenting the results. As 11 participants shared the characteristic of having formulated a wild card about some form of assisted dying, we decided to explore these participants’ reasoning and prioritizations of DöBra cards. We were curious as to whether this commonality in their use of a wild card also meant that they shared common ways of reasoning and similar prioritizations of other cards. We therefore performed more in-depth analysis of the data derived from these 11 participants, as discussed and motivated below. Four participants have been highlighted as exemplars, as each individual illustrated relatively consistent reasoning, but with clearly divergent foci when compared with one another.

Through the above analytic steps, a total of 22 interviews with 26 participants were analyzed. In the final step of analysis, we returned to the full data set to ensure that data in remaining interviews did not alter the conclusions drawn.

In presenting the findings we use exemplifying quotes to clarify analytic points, with pseudonyms used for all participants. Pauses are indicated by “. . .”, omitted phrases indicated by “[. . .]” and “[]” indicate authors’ comments.

## Results

Demographic characteristics of the 26 participants whose data were in-depth analyzed in this study and participants in the full database are reported in Table 1.

**Table 1.** Characteristics of Subsample and Full Database

Characteristics	Subsample (*n* = 26) *n* (%)	Full database (*n* = 65) *n* (%)
Age (years), median (range)	71 (43–92)	74 (43–95)
Sex		
Female	17 (65.4)	46 (70.8)
Male	9 (34.6)	19 (29.2)
Education		
University	14 (53.8)	32 (49.2)
High school	6 (23.1)	13 (20.0)
Elementary	5 (19.2)	15 (23.1)
Other	1 (3.8)	5 (7.7)
Residence		
Urban	21 (80.8)	50 (76.9)
Small town	3 (11.5)	12 (18.5)
Rural	2 (7.7)	3 (4.6)
Self-reported health status		
Good	20 (76.9)	49 (75.4)
Neither good or bad	4 (15.4)	14 (21.5)
Bad	2 (7.7)	2 (3.1)

When exploring how these participants considered and discussed their values and preferences for future EoL care in both an open conversation and semistructured interview component using the DöBra cards, we found some commonalities described in a number of broad and general descriptions of EoL values and preferences. However, these interviews were mainly characterized by detailed reasoning with notable individual variation, particularly when using the DöBra cards. Even when participants’ DöBra card rankings seemed to indicate similar priorities, we found that participants could explain why an issue was important to them quite differently. We present our findings by first describing commonalities to contextualize the findings and then elaborating on the individual nature of reasoning about EoL preferences.

### Common Patterns

When speaking in general about their future dying and death, these relatively healthy, older participants commonly envisioned their lives as characterized by forthcoming loss of abilities, and thus increasing dependence on others and formal health care services. This was described as a natural part of aging and nearing death, but it was also something that participants dreaded, as Ingrid described:

Yeah, I think that the process, feeling weaker and weaker and feeling like your life is running out, it’s pretty hard. And even worse, of course, if you have some disease and you know that it is eating away at you. And that there’s just one direction it can go …

The thought of losing independence in everyday activities and becoming reliant on others for help was troubling, as was feeling helpless, or like a burden to others. Wilma described her sense of reluctance, saying: “This is something that I think all people feel, that you don’t want to be a burden to anyone. In our society, that’s not what you’re supposed to be.” Participants also expressed concerns about losing cognitive function, self-determination, and ability to actualize decisions. Sally described what she called her “worst-case scenario”:

I have no desire to be a vegetable somewhere, that can’t … Now I’m still able to speak for myself a little bit but maybe you can’t later, I don’t know … And it’s probably among the worst things you can experience in that case. […] It goes without saying. You just don’t want that. And not being able to decide yourself what you are going to do and when you are going to do things, if you can do them at all …

Participants were used to having a sense of agency in their lives and hoped it would remain intact as long as they lived. Some spoke of wishes to be able to decide how and when they would die, and commonly expressed desires included for death to occur suddenly and unexpectedly, in the midst of everyday life. This was often said to be preferred as participants would then be able to live in their usual, independent manner to the end, avoiding loss of abilities and independence.

### Individual Patterns

Individual differences in reasoning became particularly apparent when using the DöBra cards, as they seemed to stimulate participants’ visualization of actual future situations where card statements would be relevant, thus providing context for their prioritizations. Participants could sometimes also problematize these imagined situations, envisioning them from different angles. Martin exemplified this when considering the importance of the card item “to die at home,” saying: “To die at home. No [puts card in the “not important” pile]. It kind of depends, if I am alone then I would much rather die in a [nursing] home.”

Participants could also interpret the card items as problems to find solutions for—not responding to the item itself but reflecting upon how to deal with the underlying problem. For example, Ingrid spoke about the item “to be free of pain” by including possible solutions, which seemed to prompt her to not prioritize the item as most important: “To be free of pain, I can imagine that’s something that’s in the middle [somewhat important]. One can get … Generally, there is something to take for the pain.” Thus, the DöBra cards seemed to contextualize the conversation and stimulate individually based reasoning.

### DöBra Wild Cards

The DöBra wild cards were used by 22 of 65 participants in the full database (for further detail on wild card formulations, see Tishelman et al., 2019). Eleven of these formulated their wild card to express a preference for some form of assisted dying—which is not legal in any form in Sweden—or a closely related formulation. Below we use data from these participants, whose wild card preference acts as a common denominator to demonstrate how, even when preferences were seemingly similar, EoL considerations still could differ by individual. We use the cases of Louise, Marie, Peter, and Thomas as exemplars, to contrast their manners of reasoning (see Table 2 for background characteristics).

**Table 2.** Characteristics of Exemplars

Name	Age	Residence	Self-rated health status
Louise	79	Rural	Good
Marie	66	Urban	Bad
Thomas	74	Small town	Good
Peter	72	Urban	Good

**Figure 1.**
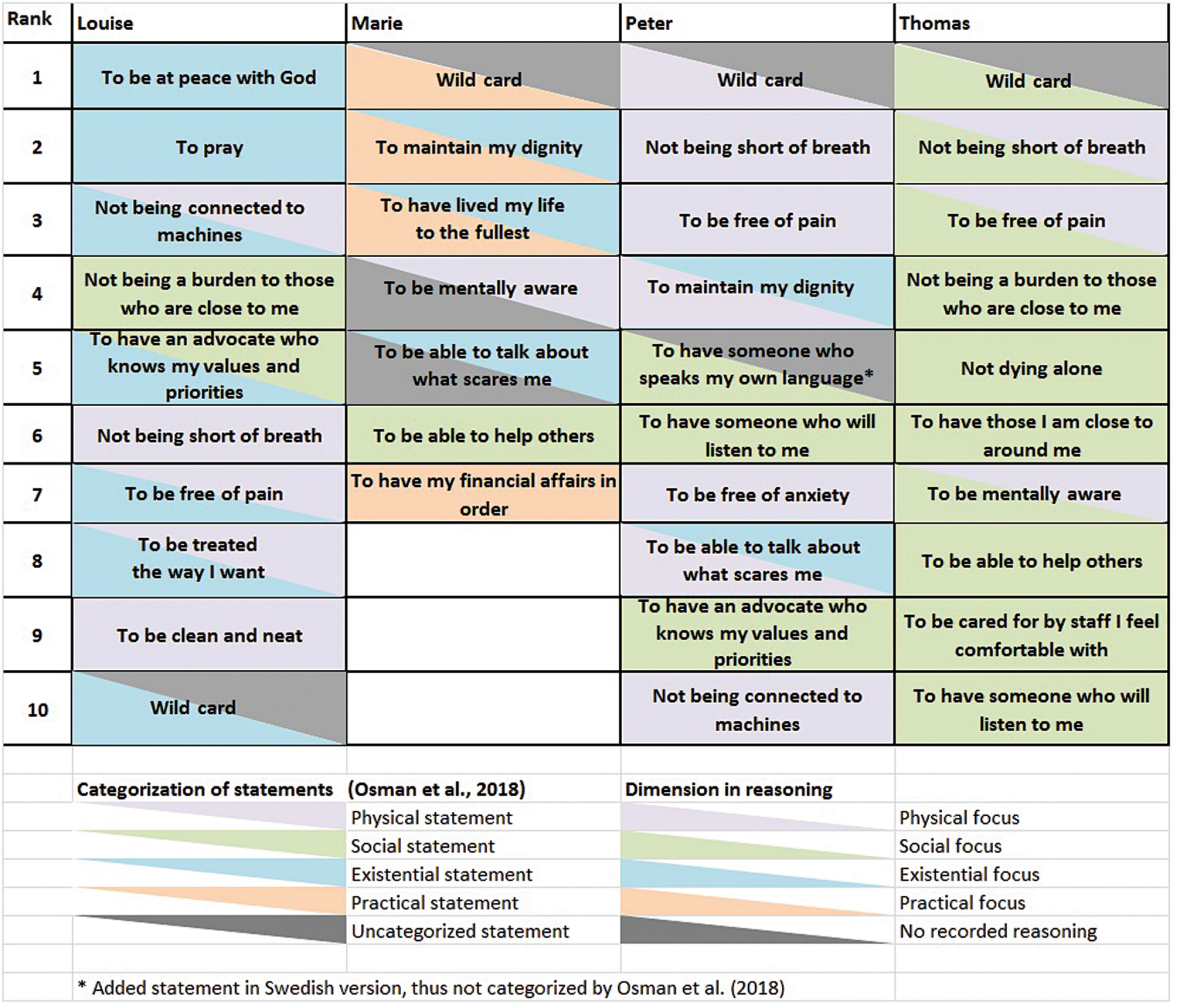
Dimensions of statements and reasoning.

When exploring how the exemplars discussed their wild card about assisted death, we found that Marie, Louise, Peter, and Thomas each considered and reasoned by tending to focus primarily on either physical, social, existential/spiritual, or practical issues. Their similarly formulated wild cards were the only preference all four shared among their top 10 priorities, as shown in Figure 1.

Quotes exemplifying how each person reasoned about their individually formulated wild card related to assisted dying are given in Table 3. Peter spoke extensively about the physical experience of dying and his dread of both being in pain and having anxiety. He also referred to his prior experience with shortness of breath, describing assisted death as a way to avoid the physical symptoms he feared. Thomas, on the other hand, while recognizing that the dying process might be difficult for him, was more concerned with how his deterioration would affect his family and his rationale for assisted death was thus to avoid suffering for those close to him. Louise spoke about existential aspects of assisted death, arguing that it should not be considered suicide, comparing it instead to the natural death of plants that wither. She also repeatedly referred to different belief systems, for example, Hinduism and Rudolf Steiner’s anthroposophical philosophy. In contrast, as illustrated in Table 3, Marie reasoned rather pragmatically about assisted death, as she matter-of-factly considered the conditions in which she felt it would be best for her life to end.

**Table 3.** Reasoning about wild cards

Participant	Primary dimension of reasoning	Exemplifying quote
Louise	Existential/Spiritual	“Yes, for my wild card people shouldn’t see it as suicide but more like something from old sagas, in Hinduism and all sorts of things. When you feel that the end is coming and your body is ready, then you can be allowed to wilt. And you don’t water a flower that is wilting … And I also know there is an idea in anthroposophy [philosophy based on work of Rudolf Steiner] that it is important for the other world that people live as fully as possible for as long as possible. But I don’t believe that, because we’re living so very artificially.”
Marie	Practical	“Yes, I’m kind of in favor of active euthanasia … I think that when I feel that I’ve given everything I can … And I kind of think … that was the limit … When I no longer know what my name is and where I live… And yeah, I think that, you know, that when you can’t fill in your own forms and all that, then you’ve lost your human dignity … when you’ve done your part, then you let it come to an end and there’s not much more to it … kind of like being used for heating [during the cremation process], I definitely wouldn’t have anything against that [body being made useful after death]*”*
Peter	Physical	“And so I think, if you’re going to lay there and really have a terrible time breathing for a longer period of time and so … that terrifies me. So in that kind of situation, I would like to say that ‘there looks like there’s no prospect of improving, so therefore I’d like to end it now’. That’s the way I would want to it to be for me.”
Thomas	Social	“Considering those close to me … I would want them to avoid having to see me as a vegetable that suffers and have the frustration that you can feel … Maybe it’s because I’ve experienced close up people who have died … where it’s been a long, drawn out process of suffering and it’s plagued the person in question and those who were close … So I’ve made up my mind that it would have been better for the person in question and those who cared about them if you had had the right to end it.”

### DöBra Card Statements

As each of the exemplars’ reasoning about their seemingly similar wild cards differed, we continued our analysis by exploring the reasoning underlying their other prioritized card items. This is illustrated in Figure 1, using two triangles for each card statement. The triangle on the upper-right represents the card dimension according to Osman and colleagues’ (2018) categorization, while the lower-left triangle represents our categorization of the participant’s reasoning about the statement. A gray triangle represents either no categorization of card statement by Osman and colleagues (2018) (i.e., for wild cards and card statements added in the Swedish version), or no recorded reasoning about this item. When the entire rectangle is of the same color, Osman and colleagues’ (2018) categorization of the statement dimension matches our interpretation of the participant’s reasoning.

As becomes evident in Figure 1, Thomas’ and Peter’s card rankings are most similar at first glance, with their top three prioritizations ranked identically. Among his 10 most prioritized cards, Thomas chose a majority of statements which Osman and colleagues (2018) categorized as socially related (six cards), whereas Peter had a more varied mix, with a predominance of cards categorized by Osman and colleagues (2018) as focused on physical aspects.

When also exploring how each individual reasoned about their prioritized card items, Thomas’ focus on social issues became even more evident as we found he reasoned with a relational focus about all of his prioritized cards, regardless of how they were categorized by Osman and colleagues (2018). When he considered the card item “To be mentally aware,” categorized by Osman and colleagues (2018) as representing the physical dimension, he expressed concern for those close to him, as he said: “Yeah, it’s simply because … if I’m completely senile … then I’m not a functioning person who can have meaningful contact with either [names partner] *or my son or my daughter, so to speak.”* Peter, in turn, reasoned about two cards that Osman and colleagues (2018) categorized as existential (Figure 1), by focusing on their physical implications. For example, when he discussed the card statement “To maintain my dignity,” Peter explained: “Yeah, that [dignity], is hard to maintain if I’m in pain. But now when I am pain-free and all, I want to be treated with dignity.” Marie’s less common focus—a more practical orientation—was evident in her discussion of the same card item about dignity, as she said:

First people buy a really expensive coffin and then a cemetery worker comes and just “dunk” [hits her fist on the table] and so it’s broken. I mean that … that’s not a dignified death, it’s just … Well, they ease their own consciences as relatives.

Marie also displayed her pragmatic reasoning when she reflected on the, for Osman and colleagues (2018), existential card item, “To have lived my life to the fullest,” by considering different options for care: “But I absolutely don’t want to be at some kind of … well, old age home or … […] I think it’s best when you have done your part, then you end it all, there’s not much more to it.” Louise, on the other hand, prioritized only two cards categorized by Osman and colleagues (2018) as existential/spiritual in her top 10 ranking, but reasoned to some extent existentially about four additional card items. For example, when reflecting on the card “Not being connected to machines,” categorized in the physical dimension by Osman and colleagues (2018), she discussed what kind of life she would consider as worth living, saying:

And not being connected to medical devices is very important to me, if it is only for life extension, that is. If it’s to save me so I can have a life worth living and I’m the one deciding what is worth living, then they can connect me to something. So, it’s a difficult decision. But if I’m unconscious they can just let me continue to be unconscious.

Participants could sometimes combine different dimensions as they reasoned. When Louise considered the card statement categorized by Osman and colleagues (2018) as physical, “To be treated the way I want,” she reasoned about both her physical and existential needs, as she said:

Do I want them to sit and hold my hand or not? I would very much like for [names daughter] to hold my hand, if she wasn’t also still holding me alive, but if in her thoughts she was asking me to let go and move on.

As shown above, different participants could have a different predominant focus in their ways of reasoning, even when considering the same card items. This was also evident in Louise’s, Peter’s, and Thomas’ reflections on the card item, “To be free of pain,” which Osman and colleagues (2018) categorize as representing the physical dimension. As given in Table 4, Peter again demonstrated his clear focus on the physical which dominated much of his reasoning, while Thomas continued his focus on social matters, and Louise again related to existential issues. There is clearly more to a card statement than what first meets the eye and understanding why a DöBra card item is important to an individual may be at least as important as the level of prioritization of the card statement itself.

**Table 4.** Different Reasoning About the Same Card Item

Participant	Primary dimension of reasoning	Exemplifying quote about card item *“To be free of pain”*
Louise	Existential/Spiritual	“Yes, “to be free of pain” is important. The anthroposophists [a philosophy based on Rudolf Steiner’s work] think that suffering is part of life, and those who believe whole-heartedly in anthroposophy, they won’t even agree to get a lot of pain relief. But my mother got, she actually took morphine when she got to the point … And that’s what I’ll do. No one has to be stingy about it, just pile it on. Even if it shortens life by a few hours or a few days, it’s totally ok. I want to die free from pain.”
Peter	Physical	“But for my part, if I’m in that kind of situation [with unendurable pain], *then I would prefer having help to die … And have that definitive life experience, rather than dying in pain* [laughs].*”*
Thomas	Social	“If I have such unbearable pain so that I can’t stand it, then you can’t have meaningful relationships with one another either, so to speak.”

## Discussion

In this article, we explore commonalities and individual differences in how people reason about what they consider important to them in their future EoL. We do this by analyzing data from 65 community-dwelling older adults engaged in ACP conversations in the otherwise ACP-naive Swedish setting. We found that values common to this group were in line with those from existing literature (Caswell & O’Connor, 2019; Robinson and colleagues, 2019), including desires to remain independent even at the EoL, to maintain agency in one’s life situation, and not to be a burden to others. However, a significant contribution of this study is showing ways in which the individual nature of reasoning about EoL preferences became particularly evident when using the DöBra cards, as the card statements seemed to trigger more situation-based reasoning in which participants imagined and problematized a future situation and their own preferences related to it. Further individual differences were found when exploring how participants’ reasoning about the same card statements varied, as exemplified by Louise, Marie, Peter, and Thomas, who despite similarities in cards prioritized, differed in how they reasoned about their choices.

While we were inspired in the analytic phase by Osman and colleagues’ (2018) differentiation of GoWish statements into four different dimensions, our findings led us to problematize this predetermined form of categorization. We found that Osman and colleagues’ (2018) categorization of each card item into one of the four particular dimensions did not adequately reflect the flexibility of the card items. Our analysis indicated that the cards allowed great variation in how participants could reason about them. Another criticism that can be directed toward the a priori categorizations by Osman and colleagues (2018) and Steinhauser and colleagues (2000) is that there is no reason to believe that a set number of dimensions is exhaustive—in theory, there may be additional kinds of underlying values steering individual preferences, for example, ethical or aesthetic reasoning. We emphasize that our analytic intention has been limited to pointing to the individual nature of reasoning, rather than attempting to compile a comprehensive list of possible manners of reasoning.

These findings regarding variation in underlying reasoning when using the DöBra cards do, however, point to a strength of our Swedish DöBra cards, and of the original GoWish cards. The card statements have been formulated broadly enough to support specificity in reasoning about EoL preferences without necessarily steering the conversation in a predetermined direction and allow for reasoning about underlying values which may be culture- and context-specific. Considerations motivating the choice of a specific card in many cases appeared to be quite independent of the apparent face value of the card statement, as exemplified in the data presented here. One conclusion which can be drawn is that the sorting of the cards according to level of prioritization is only one of the benefits of using the cards—how the cards support a substantial discussion of what is important to the individual at the EoL may be even more valuable. This conclusion is similar to that of Potthoff and colleagues (2017), who used GoWish cards with parents of critically ill children in a pilot study, concluding that parents felt empowered to discuss EoL issues as the cards helped them concretize their thoughts. The DöBra cards also seemed to stimulate the discussion to be more focused on what participants actually *did want*, as opposed to more generally formulated wishes in the initial conversational part of the interview, which often focused on what participants *did not want*, that is, not wanting to lose abilities, independence, and not wanting to be a burden to others.

EoL preferences were sometimes discussed not only in terms of what would be best for the participant alone, but also related to what the participants felt would be best for those close to them, in line with results of studies conducted in other contexts (Clarke & Seymour, 2010; Liu & van Schalkwyk, 2019). In general, participants seemed not to view death itself as their greatest concern for the future, instead speaking of fearing dependence on others coupled with their loss of independence, as found also in other studies of EoL preferences of both older and younger people (Caswell & O’Connor, 2019; Lloyd-Williams, Kennedy, Sixsmith, & Sixsmith, 2007; Robinson et al., 2019). As noted by Caswell and O’Connor (2019), some participants reacted to the threat of losing independence by contemplating ways in which to take control of the situation. Medical assistance in dying was discussed as a means of taking control in the present study, as well as by Caswell and O’Connor (2019). The frequency with which this was discussed in our study was particularly notable because assisted dying is not legal in any form in Sweden and may be perceived as difficult to discuss. In our previous research, for example, we found that the subject of assisted death was never raised in group discussions (Tishelman et al., 2019).

While discussions of assisted dying were relatively common in this study, our sampling with active volunteering should be considered, as it might have led to participants who had an explicit interest in these matters to be more prone to volunteer. While some might argue for a preformulated statement in the GoWish/DöBra card decks concerning assisted dying, we would instead argue the benefit of wild cards for users to freely formulate individual wishes. The range of wild card formulations concerning assisted dying and the variety in underlying reasoning, in combination with the legal status of euthanasia in Sweden and the risk of being unduly provocative for many, further strengthens our position not to add a preformulated item about assisted dying to the DöBra cards.

Our sampling strategy also inhibits broad generalizations to other populations; however, we would argue that the present study offers an important contribution to knowledge about the reasoning and considerations leading to prioritized EoL preferences. While the GoWish cards have been used in several recent studies of different contexts and populations (Banner et al., 2019; Delgado-Guay et al., 2016; Glennon et al., 2019; Kuramoto et al., 2015; Zachariah, Klein, Clifton-Hawkins, Andrews, & Gross, 2015), we have found few other studies reporting on how GoWish items have been reasoned about and negotiated by users. The study by Lee, Hinderer, and Alexander (2018) on EoL preferences for Chinese Americans is a rare exception, also involving community-dwelling older adults, as they briefly report on how participants commented card items. While reasoning and prioritizations of the card item “Not being a burden to my family/those close to me” seem similar in both studies, consideration of the card item “Being free of pain” has notable differences, as the study by Lee and colleagues (2018) reports only on reasoning about the physical experience of pain, whereas we have found different underlying ways of reasoning for prioritizing this and other card items among different individuals. Furthermore, as no wild cards were used in the study by Lee and colleagues (2018), and are rarely reported in other studies (Lankarani-Fard et al., 2010; Litzelman et al., 2017), our in-depth descriptions of how participants have reasoned when considering the wild cards are of value for understanding the very individual nature of prioritization of EoL preferences.

### Implications

We found that the DöBra cards are a promising tool to support person-centered conversations about EoL preferences, in this study both for individuals and couples. The present study particularly highlights the importance of the conversation and reasoning surrounding the card sorting. The ranking of cards alone, though an engaging conversation starter, is not sufficient means to comprehensively understand an individual’s values and preferences for future EoL care. We therefore suggest that future users of the DöBra/GoWish cards, regardless of setting, also consider the reasoning underlying an individual’s prioritizations to strengthen person-centeredness in EoL conversations and in care situations. Understanding individuals’ underlying values can provide understanding to guide EoL decision making by individuals, families, and care providers, not least when individuals can no longer speak for themselves.

## Supplementary Material

gnaa012_suppl_Supplementary_Material

## Footnotes

1 DöBra is a Swedish pun, meaning both “dying well” but also “awesome.”
