# Supplementary material for: Exploring Community-Dwelling Older Adults’ Considerations About Values and Preferences for Future End-of-Life Care: A Study from Sweden
Source: Gerontologist. 2020 Mar 21;60(7):1332–42. doi: 10.1093/geront/gnaa012 (PMC7491437; doi:10.1093/geront/gnaa012)
Supplement: gnaa012_suppl_Supplementary_Material [file gnaa012_suppl_supplementary_material.docx]

# Supplementary Material

*DöBra card statements^a^ translated to English*

| \| **DöBra card statement** \| \| --- \| \| To be free of pain \| \| Not being short of breath \| \| To be clean and neat*^b^* \| \| To be free of anxiety \| \| To have a human touch \| \| To have those who are close to me be prepared for my death*^b^* \| \| To die at home \| \| To say goodbye to important people in my life \| \| To remember personal accomplishments \| \| To take care of unfinished business with others*^b^* \| \| To be treated the way I want \| \| To maintain my dignity \| \| To keep my sense of humor \| \| To have close friends near \| \| To have someone who will listen to me \| \| Not being a burden to those who are close to me*^b^* \| \| To be able to help others \| \| To be able to talk about what scares me \| \| To have those I am close to around me*^b^* \| \| To have lived my life to the fullest*^b^* \| \| To have a doctor who knows me well*^b^* \| \| Not dying alone \| \| To be mentally aware \| \| To pray \| \| To meet with a representative of my religion \| \| To be able to talk about what death means \| \| To be at peace with God \| \| To have my financial affairs in order \| \| To know how my body will change \| \| To prevent arguments by making sure that those close to me know what I want*^b^* \| \| To have an advocate who knows my values and priorities \| \| To trust my doctor \| \| To be cared for by staff I feel comfortable with a \| \| To have my funeral arrangements made \| \| Not being connected to machines \| \| WILD CARD \| \| To have someone who speaks my own language (New card) \| \| To know how my body will be taken care of after death (New card) \|   *^a^ The DöBra card statements originate from the American GoWish cards, but have been adapted for use in Sweden, with permission from originators (www.codaalliance.org)*  *^b^ Statement has been culturally adapted to the Swedish language and context* |
| --- | --- | --- | --- | --- | --- | --- | --- | --- | --- | --- | --- | --- | --- | --- | --- | --- | --- | --- | --- | --- | --- | --- | --- | --- | --- | --- | --- | --- | --- | --- | --- | --- | --- | --- | --- | --- | --- | --- | --- |

**Interview guide**

This interview guide presents topics to be covered in the interview, and the moderator will ask probing questions during the participants’ stories

Examples of probing questions:

- Can you tell me more about that?
- What do you mean?
- What are your thoughts about that?

**1. Initial, conversational component**

- Please tell me why you were interested to participate in this study.
- Participants will be asked to talk about:
  - Their own thoughts about what would be important to them when life is coming to an end.
  - Who they think would be important to them at the end-of-life
  - If they have discussed their preferences for EoL with anyone else

**2. Structured component**

- EcoMapping
  - Each participant places themselves in the middle of the map and writes down important people or groups around, e.g. family, friends, colleagues, informal groups, formal organizations, health care providers. Lines are drawn from themselves to each contact on the map. 1 line = weak relationship, 2 lines = moderate, 4 lines = strong relationship. A wavy line may be drawn to depict tensions in a relationship.
  - Participants will be asked for their reflections on their social network and about drawing the EcoMap.
- DöBra cards
  - Participants will sort the statements in the card deck into three different piles according to level of importance to them: very important, somewhat important and not important. They will be asked to share any reflections they have as they go through the card deck. They will be asked if they want to add a wild card for other matters of importance to them which are not covered by the pre-formulated items. Participants till then rank their 10 most important cards from the ‘very important pile’ with the 1^st^ statement being the highest priority.
  - Participants will be asked to speak aloud about why they think that each of the 10 most important statements is important.
  - Participants will be asked about their reflections on using the DöBra cards.

**3. Demographic information**

*Sex:*

*Age:*

*Place of residence:*

*Country of birth?*

□ Sweden

□ Other, namely…

*Living situation, lives with…*

□ wife/husband/partner/co-habitant

□ other adult

□ minor children

□ living alone

□ other

*Highest level of completed education*

□ Elementary school

□ High school

□ University/college

□ Other

*Employment status*

□ Working full-time

□ Working part-time

□ Unemployed

□ Student

□ Retired

□ On sick leave

□ Other, namely…

What is/has been your main occupation?

……………………………..

*Self-rated health status: How do you experience your health status today?*

□ Good

□ Neither good nor bad

□ Bad

**4. Ending the interview**

- How did you experience our conversation about these matters?
- Is there anything else regarding the EoL that we have not discussed, which you would like to raise?
- May we contact you again in 6-12 months for a follow-up interview?

Participants will be reminded that they are welcome to contact us if they have any questions, comments or reflections, and will be given contact details.
